# Fingertip advanced glycation end products and psychotic symptoms among adolescents

**DOI:** 10.1038/s41537-021-00167-y

**Published:** 2021-08-12

**Authors:** Mitsuhiro Miyashita, Syudo Yamasaki, Shuntaro Ando, Kazuhiro Suzuki, Kazuya Toriumi, Yasue Horiuchi, Akane Yoshikawa, Atsushi Imai, Yukihiro Nagase, Yasuhiro Miyano, Tomoko Inoue, Kaori Endo, Yuko Morimoto, Masaya Morita, Tomoki Kiyono, Satoshi Usami, Yuji Okazaki, Toshiaki A. Furukawa, Mariko Hiraiwa-Hasegawa, Masanari Itokawa, Kiyoto Kasai, Atsushi Nishida, Makoto Arai

**Affiliations:** 1 Schizophrenia Research Project, Department of Psychiatry and Behavioral Sciences, Tokyo Metropolitan Institute of Medical Science, Tokyo, Japan; 2 Department of Psychiatry, Tokyo Metropolitan Matsuzawa Hospital, Tokyo, Japan; 3 Department of Psychiatry, Takatsuki Clinic, Akishima, Japan; 4 Department of Psychiatry, Takatsuki Hospital, Hachioji, Japan; 5 Research Center for Social Science & Medicine, Tokyo Metropolitan Institute of Medical Science, Tokyo, Japan; 6 Department of Neuropsychiatry, Graduate School of Medicine, The University of Tokyo, Tokyo, Japan; 7 Department of Psychiatry, Shinshu University School of Medicine, Matsumoto, Japan; 8 Niigata University Graduate School of Medical and Dental Science, Niigata, Japan; 9 Center for Research and Development on Transition from Secondary to Higher Education, The University of Tokyo, Tokyo, Japan; 10 Health Promotion & Human Behavior, Kyoto University School of Public Health, Kyoto, Japan; 11 Department of Evolutionary Studies of Biosystems, The Graduate University for the Advanced Studies, SOKENDAI, Hayama, Japan; 12 The International Research Center for Neurointelligence (WPI-IRCN) at The University of Tokyo Institutes for Advanced Study (UTIAS), University of Tokyo, Tokyo, Japan

**Keywords:** Psychosis, Schizophrenia, Molecular neuroscience, Developmental biology

## Abstract

Case control studies have suggested that advanced glycation end products play a key role in the pathophysiology of chronic schizophrenia. However, the longitudinal association between advanced glycation end products and psychotic symptoms among drug-naïve adolescents remains unclear. This study examined whether advanced glycation end products could predict the trajectory of psychotic symptoms in drug-naive adolescents using data from prospective population-based biomarker subsample study of the Tokyo Teen Cohort. A total of 277 community-dwelling adolescents aged 13 years without antipsychotic medication were analyzed. Fingertip advanced glycation end products were measured in adolescents using noninvasive technology that can be used quickly. The trajectory of psychotic symptoms in a 12-month follow-up was assessed by experienced psychiatrists using a semi-structured interview. Of the 277 participants, 13 (4.7%) experienced persistent psychotic symptoms (psychotic symptoms at baseline and follow-up), 65 (23.5%) experienced transient psychotic symptoms (psychotic symptoms at baseline or follow-up), and 199 (71.8%) did not have psychotic symptoms. Multinomial logistic regression analysis adjusted for age and sex revealed that baseline fingertip advanced glycation end products might predict the risk of persistent psychotic symptoms (odds ratio = 1.68; 95% confidence interval, 1.05–2.69; *P* = 0.03). Altogether, fingertip advanced glycation end products potentially predicted the trajectory of psychotic symptoms among drug-naive adolescents, which indicated its involvement in the pathophysiology of early psychosis. Further studies are required to identify strategies to reduce adolescent advanced glycation end products, which may contribute to preventing the onset of psychosis.

## Introduction

Advanced glycation end products (AGEs), defined as irreversible products derived from the nonenzymatic reaction of reducing sugars with proteins and amino acids, have been suggested to play a key role in the pathophysiology of adult psychosis, including schizophrenia. A number of case control studies have shown that accumulation of plasma AGEs are associated with schizophrenia according to our previous report^1,2^. A recent study indicated that AGEs affect brain volume in patients with recent-onset psychosis^3^. Brain inflammation with AGEs accumulation could be a potential cause of the development of psychotic symptoms^4^. AGEs accelerate the brain inflammation by binding the membrane-bound receptor for AGEs (RAGE)^5,6^, and the blockade of AGEs-RAGE interaction inhibits the inflammation. Increase of AGEs^1,2^ and decrease of anti-AGEs markers (e.g., vitamin B6^1,2,7^ and soluble receptor for AGEs^8^) have been found among patients with psychotic disorders. Furthermore, another study showed that a novel biological therapy, aimed at reducing AGEs, demonstrated clinical improvement of psychotic symptoms in adult schizophrenia patients^9,10^ These findings suggest that AGEs are implicated in the etiology of psychosis. However, a longitudinal association between AGEs and psychotic symptoms has not yet been established, and the effect of antipsychotic medication on increased AGE levels cannot be ruled out in previous studies. To address these issues, prospective cohort studies on drug-naïve subjects are required.

Adolescent psychotic symptoms have been associated with the risk of mental disorders^11^, specific transition to schizophrenia spectrum disorder^12,13^, illness progression^14^, higher suicide rate^15–17^, and metabolic abnormality^18^. While the prevalence of adolescent psychotic symptoms is relatively high, only a small proportion develop psychotic disorder^19^. Therefore, recent studies have focused on the persistence of psychotic symptoms to identify adolescents at high risk for the onset of psychosis^20^. However, to the best of our knowledge, only a few studies have examined the trajectory of psychotic symptoms in drug-naïve adolescents. To investigate the role of AGEs in the onset of psychosis, it is a unique opportunity to investigate whether AGEs predict persistent psychotic symptoms among drug-naive adolescents.

Meerwaldt et al. first reported on the use of skin autofluorescence (SAF)^21^, a noninvasive AGE measurement, which uses the characteristic fluorescence spectrum of AGEs. Since then, several cross-sectional studies indicated that increased SAF was associated with schizophrenia^22^ and recent-onset psychosis^23^. Yamanaka et al. successfully developed an advanced SAF reader using the tip of the middle finger of the nondominant hand, to exclude the influence of skin pigmentation on SAF^24^. They also confirmed that the intensity of fingertip AGEs positively correlated with serum AGEs^24^. Thus, fingertip autofluorescence (FAF) reader offers a great advantage in terms of a noninvasive and precise method for AGE measurements especially for adolescents.

Therefore, this study aimed to examine whether AGEs among drug-naïve adolescents could predict the trajectory of psychotic symptoms using noninvasive AGE measurement.

## Results

### Characteristics of participants

Table 1 shows the characteristics of adolescents who completed the study including participants who had taken neuroleptic medication or not. There were no significant differences in sex, socioeconomic status, and psychotic symptoms at baseline (*P* > 0.05) between participants who were successfully followed and those who dropped out from the follow-up survey, although those lost to follow-up were older than those who completed the follow-up (mean [SD] 13.6 [0.6] vs. 13.4 [0.6] years at baseline, *P* = 0.02). We confirmed that there were no smokers or patients with diabetes among the participants. We also found no associations between baseline SAF and urine creatinine levels, which reflect renal function (*r* = 0.007, *p* = 0.902) among participants, as well as no group differences in urine creatinine levels among the three trajectory groups (see Table 1) in our study. Of the 282 participants, five participants have already received neuroleptic medication. Among drug-naive adolescents, 13 (4.7%) were assessed as presenting with persistent psychotic symptoms (assessed as psychotic symptoms at baseline and follow-up) during the 12-month follow-up. Among the rest of the sample, 199 (71.8%) and 65 (23.5%) participants had no psychotic symptoms and transient psychotic symptoms, respectively.

**Table 1:** Characteristics of participants in the general adolescent cohort.

	All participants	No psychotic symptoms^a,b^	Transient psychotic symptoms^a,c^	Persistent psychotic symptoms^a,d^	*p*
*N* (%)	282 (100.0)	200 (70.9)	67 (23.8)	15 (5.3)	–
Age at baseline (years, mean [SD])	13.4 [0.6]	13.4 [0.5]	13.5 [0.6]	13.4 [0.5]	0.866^e^
Sex (male/female, *N*)	156/126	108/92	39/28	9/6	0.779^f^
Fingertip AGEs at baseline (a.u., mean [SD])	0.44 [0.06]	0.44 [0.06]^g^	0.44 [0.07]	0.48 [0.09]^g^	0.040^g^
Urine creatinine levels (mg/dl, mean [SD])	153.2 [67.5]	151.8 [59.7]	157.0 [78.4]	154.9 [106.5]	0.862^e^
Lower socioeconomic status^h^, *N* (%)	24 (8.9)	16 (8.4)	7 (10.8)	1 (7.1)	0.819^f^
Parental history of psychiatric disorders, *N* (%)	10 (3.5)	6 (3.0)	4 (6.0)	0 (0.0)	0.391^f^

*AGEs* Advanced glycation end products, *a.u.* arbitrary unit, *SD* standard deviation.

^a^Psychotic symptoms evaluated using the Scales for the Assessment of Positive Symptoms. Follow-up assessment was administered 1 year from baseline.

^b^Cases without psychotic symptoms both at baseline and at follow-up.

^c^Cases with psychotic symptoms only at baseline or at follow-up.

^d^Cases with psychotic symptoms both at baseline and at follow-up.

^e^Analysis of variance test for group differences.

^f^Chi-square test for group differences.

^g^Tukey’s multiple comparison test showed significant difference between no psychotic symptoms group and persistent psychotic symptoms group.

^h^Family annual income was lower than 4 million yen at age 12.

### Association between fingertip AGEs and trajectories of psychotic symptoms

**Fig. 1:**
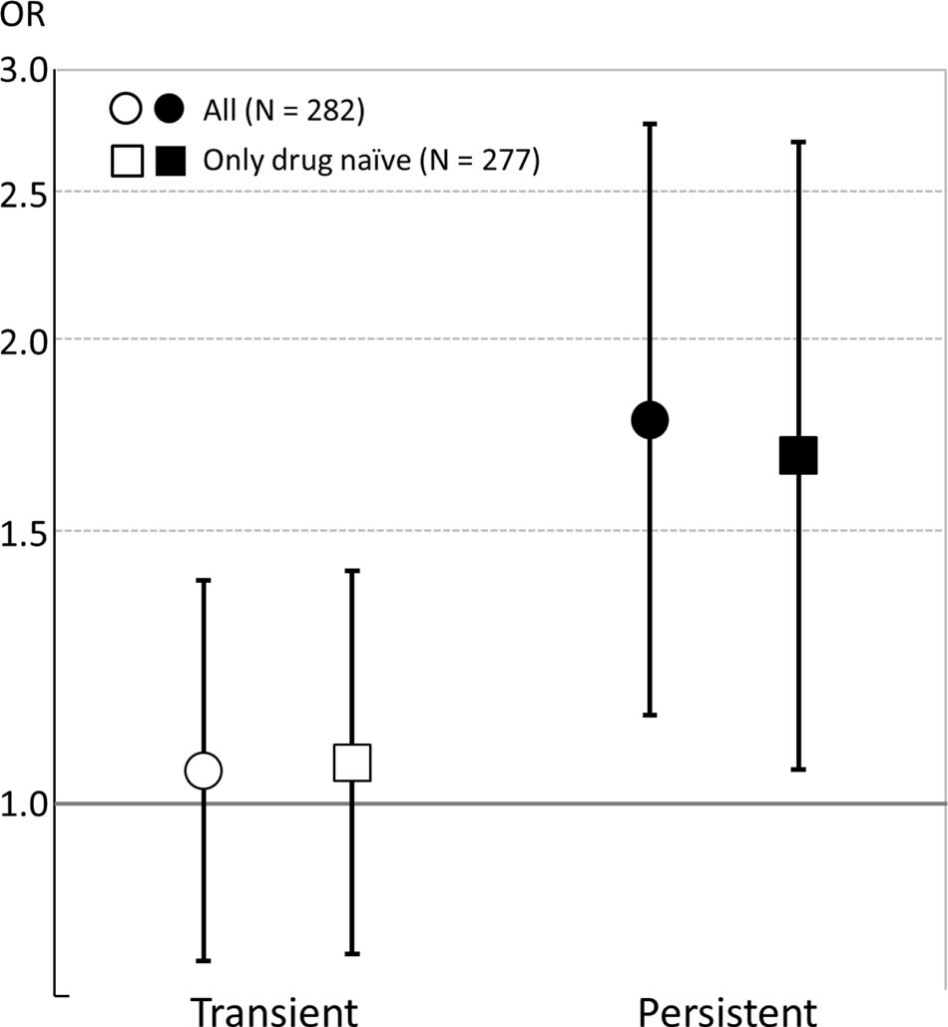
The association between fingertip advanced glycation end products and the trajectory of psychotic symptoms. Odds ratios (OR) for standardized scores of fingertip AGEs from multinomial logistic regression analysis potentially predicting persistence of psychotic symptoms for 1 year among adolescents (reference: no psychotic symptoms at baseline and follow-up) adjusting for age at baseline and sex. Error bars indicate the 95% confidence interval.

As shown in Fig. 1, multinomial logistic regression analysis adjusted for age and sex demonstrated that ORs for persistent and transient psychotic symptoms compared with those for no psychotic symptoms were 1.78 (95% CI, 1.14–2.76, *P* = 0.011) and 1.05 (95% CI, 0.79–1.40, *P* = 0.73), respectively, for a 1SD increase in fingertip AGEs. The significance and magnitude of effect size for persistent psychotic symptoms remained even after excluding five participants who were using antipsychotic medications (OR, 1.68; 95% CI, 1.05–2.69; *P* = 0.03; Fig. 1). Similar results were confirmed when we excluded the cases who experienced psychotic symptoms only at follow-up (*N* = 5) from the transient group (ORs for persistent and transient psychotic symptoms were 1.72 [95% CI, 1.07–2.78, *P* = 0.027] and 1.16 [95% CI 0.86–1.56, *P* = 0.337], respectively.

## Discussion

In a 12-month longitudinal study of drug-naive adolescents in the general population, we have shown that baseline fingertip AGEs could differentiate persistent psychotic symptoms from no psychotic symptoms during a year. The findings of the present study have important clinical implications for the role of AGEs in the pathophysiology of early psychosis and may bring about new insight into early intervention to prevent psychosis.

Several cross-sectional studies have reported that increased AGE levels are associated with adult psychosis^1,2^, however, the direction of the relationship is unknown as there has been no longitudinal study. Although the influence of antipsychotic use on the accumulation of AGEs has been controversial^23,25,26^, antipsychotic medication could be a potential confounder in the increase of AGEs in schizophrenia. We overcame these concerns by revealing the prospective association between increased AGE levels and persistent psychotic symptoms among drug-naive adolescents. Moreover, prolonged psychotic symptoms are a well-known risk factor for transition to psychosis. This suggests that AGEs are involved in the process of psychosis development.

In general, the accumulation of AGEs occur under the condition of long-term excess glucose, which is a source of AGEs. It is also well known that stress induces hyperglycemia via activation of the hypothalamic-pituitary-adrenal axis^27^. Furthermore, chronic social defeat stress has been shown to induce long-term peripheral and central hyperglycemia and impaired glucose metabolism in a mouse model^28^. In addition, an experimental study using human neural progenitor cell lines revealed hyperglycemia-induced oxidative stress^29^, which is known to enhance the accumulation of AGEs^30^. Also, mental stress was shown to correlate with skin AGEs in a human study^31^. Although we did not measure the psychological stress in this cohort, it can be suggested that increased fingertip AGE levels in adolescents with persistent psychotic symptoms were caused by continuous hyperglycemia induced by sustained psychological stress, and the accumulation was enhanced through the synergistic effect of adverse lifestyle, such as lack of exercise^31^ or high AGE diet^32^. In the future wave of this cohort study, blood glucose levels should be measured to further investigate the relationship between psychological stress, hyperglycemia, and increase in AGEs.

The biological mechanism by which increased AGEs may cause psychotic symptoms has been discussed since the first study on the association between AGEs and schizophrenia.^1^ One explanation for the underlying mechanism is brain inflammation. This is because binding of AGEs to the RAGE, a membrane-bound receptor expressed in neurons, astrocytes, and microglia, evokes upregulation of proinflammatory cytokines^5^. Indeed, chronic stress exposure caused behavioral disturbances in a mouse model through increased RAGE expression in the microglia, which was rescued by the knockout of RAGE^33^. In human studies, genetic polymorphisms in RAGE gene was associated with schizophrenia^34^. In addition, it was reported that the plasma concentration of soluble receptors for AGEs, which inhibits inflammation due to a lack of membrane-bound domain, was decreased in patients with schizophrenia^8^. Altogether, we could suggest that brain inflammation induced by the AGE–RAGE interaction may be a possible mechanism underlying the psychotic symptoms observed in adolescents.

It is particularly important for AGEs to potentially predict persistent, but not transient, psychotic symptoms. The persistence of psychotic symptoms is a high-risk indicator of transition to psychotic disorders, such as schizophrenia^20^. Although, the sensitivity and specificity of fingertip AGEs remains to be investigated, this may allow us to interpret that fingertip AGEs may be used as a biomarker for the early identification of high-risk adolescents. In addition, fingertip AGEs are a noninvasive and easy tool that may be broadly applicable in both clinical and community settings. Furthermore, the objective physical evaluation of the measurement may have an advantage in providing a chance for intervention in those who have difficulties disclosing the mental health problems and displaying help-seeking behavior. Previous studies reported interleukin-6^35^ and p300^36^ as biological markers for the risk of the onset of psychosis; however, these measurements required invasive and complicated examinations. Thus, in the future, the usefulness of fingertip AGEs may contribute to better mental health services in clinical practice and community settings, including schools. This includes individualized psychosocial management in adolescents with increased fingertip AGE levels and development of new biological treatments to reduce AGE levels. Furthermore, promoting a healthy lifestyle, including a low AGE diet or appropriately tailored exercise, may serve as a potentially preventive platform for the transition to psychosis. In the future study, it should be tested whether the fingertip AGEs could predict the transition to psychosis

Skin AGEs are associated with cardiovascular disease^37^ and mortality in healthy control independent of glycemic measures and the metabolic syndrome^38^. As for recent-onset psychosis, relationship between skin AGEs and cardiovascular risk was also identified^23^. In addition, association between unhealthy lifestyle and worsening of cardiometabolic risk was found in FEP^39^. The results of these studies may provide the potential role of AGEs in the link between increased risk for cardiovascular disease and psychosis.

This study has several strengths. First, the population-based longitudinal study design enabled us to show that AGEs may predict persistent psychotic symptoms in adolescents. Second, the study had a high quality assessment for psychotic symptoms using semi-structured interviews conducted by experienced psychiatrists. Third, the study recruited drug-naïve adolescents to eliminate the effect of antipsychotic medications on AGEs. Fourth, the study employed the innovative technique of AGE measurement using the fingertips, which leads to quick, noninvasive, and accurate measurement of AGEs in adolescents.

One of the limitations of the present study is attrition. Eighteen percent of the participants failed to complete the study, which prevented their inclusion in the outcome analyses. However, there was no difference in psychotic symptoms between participants who completed the study and those who dropped out. The AGEs might predict persistent psychotic symptoms even after adjustment for age, and was significantly different. Thus, it is likely that bias from sample attrition did not affect the estimation of the association between AGEs and persistent psychotic symptoms in our study. In the present study, we demonstrated that baseline fingertip AGEs may predict the persistence of psychotic symptoms; however, the association between the trajectory of fingertip AGEs and psychotic symptoms, as well as whether baseline fingertip AGEs can predict future occurrence of psychotic symptoms, needs to be addressed in future research. Another limitation was the lack of blood sampling in this cohort. As mentioned above, investigation using blood samples will enable us to validate the results of this study and to gain accurate biological insights into the role of AGEs in the development of psychosis. In addition, to control potential confounding factors, detailed information about participants’ lifestyle should be used, which may affect fingertip AGE levels (e.g., food intake and exercise) in further studies.

In conclusion, the present study provides new evidence that increased AGE levels may predict persistent psychotic symptoms among drug-naive adolescents, which suggests that AGEs are involved in the processes associated with the development of psychosis. Noninvasive and easy operation of the measurement may be widely accepted in clinical and community settings, and further study is needed to investigate whether fingertip AGE measurement support young individuals to have a better life.

## Methods

### Participants

The current study was conducted as part of the population-based biomarker subsample study of the Tokyo Teen Cohort Study (TTC, http://ttcp.umin.jp/) (pb-TTC), in which 345 adolescents (mean [standard deviation, SD], 13.5 [0.6] years) and their caregivers (mainly mothers) were included. The study examined biological markers, including AGEs and psychiatric symptoms as assessed with semi-structured interviews by expert psychiatrists.

The participants in the pb-TTC were recruited from a larger sample of the TTC project, a large-scale population-based birth cohort study conducted in the Tokyo metropolitan area with >3000 adolescent-caregiver dyads^40–42^. No significant differences in age, sex, and socioeconomic status were found between the pb-TTC subsample (*N* = 345, 197 [57.1%] boys) and the other TTC population (*N* = 2826, 1487 [52.6%] boys; no differences in sex [*p* = 0.115]; mean age in months [SD] at the recent TTC survey: 145.8 [3.6] and 146.1 [3.7], *p* = 0.229; prevalence of lower socioeconomic status, which was defined by annual family income of <400 million yen: 8.9% and 9.4%, *p* = 0.799, respectively). We longitudinally followed the pb-TTC subsample after a year. Of the 345 participants in the baseline survey, 282 participants (82%) completed the follow-up survey.

Written informed assent and consent were obtained from each participant and their main caregiver before participation, respectively. The study was approved by the research ethics committees of the Tokyo Metropolitan Institute of Medical Science.

### Measurement of fingertip AGEs

Fingertip AGE levels were evaluated using an FAF reader (Sharp Life Science Corporation, Japan). The details of the measurement are described elsewhere^24^. Briefly, the middle fingertip of the nondominant hand was cleaned with alcohol cotton swab before placing it on the sensor for measurement. The FAF was assessed at an excitation wavelength of 340 nm and emission wavelength of 440 nm. To minimize the effect of body movement on the measurement, the finger was pinched with a clip at 5.5–6.0 N. The FAF measurement was performed twice; however, another measurement was added if the variation between the second and first trial was >10%. Due to the noninvasive and easy to operational nature of the measurement, almost all participants quickly completed the measurement within 3 min. Interestingly, similar to the AGE measurement using blood samples, the fingertip AGE method is also able to distinguish between adult schizophrenia patients and controls (Supplementary Table 1, Supplementary Table 2, and Supplementary Fig. 1 in the Supplementary Information).

### Measurement of outcome

Psychotic symptoms at ages 13 and 14 years were examined in a semi-structured interview by experienced psychiatrist (S.A., M.M., or K.S.). The assessment of psychotic symptoms was conducted in three stages on the same day. First, participants were asked to respond to a brief self-report questionnaire including the Adolescent Psychotic-Like Symptom Screener (APSS), which could screen the general adolescent population for psychotic-like symptoms with a high degree of accuracy^43^. It was used in previous cohort studies in Japan with sufficient reliability^42,44^. The APSS consists of seven items related to psychotic symptoms: hallucinations (visual and auditory) and delusions (mind reading, reference, being spied on, being controlled, and grandiose ability). For each question, the possible responses were “No, never”, “Maybe”, or “Yes, definitely”. Second, after completing the self-report questionnaire, all participants were interviewed by experienced psychiatrists. If the child answered “Maybe” or “Yes, definitely” to any of the seven items from the APSS, the responses were cross-examined in a semi-structured format using each of the seven related symptoms derived from the Scales for the Assessment of Positive Symptoms^45^ to obtain an observer-based rating of the symptoms. The episodes of each positive symptom and their frequency in the previous 6 months were recorded. Interviewers also ascertained whether positive symptoms were experienced when the child was in a hypnagogic and hypnopompic state, had fever, or had been drinking alcohol or using drugs. Third, after each interview, a consensus meeting was held by the three psychiatrists (S.A., M.M., and K.S.) to review each interview record and reach a consensus. Participants were rated as having “definite” psychotic symptoms without attributions if there was agreement among all three psychiatrists.

Longitudinal profiles of psychotic symptoms across the two time points (baseline, follow-up) were defined as follows:

i. No experiences: Individuals without a psychotic symptoms at any time point.
ii. Transient: Individuals with a psychotic symptoms determined at only one time point.
iii. Persistent: Individuals with a psychotic symptoms at two time points.

### Measurement of potential confounding factors

Socioeconomic status was measured by asking annual family income in the past year, and parental history of psychiatric disorders was assessed using self-reported questionnaire. Urinary creatinine levels were measured to examine renal function using the first urine sample collected in the morning at the baseline survey of the pb-TTC.

### Statistical analysis

Data are presented as mean [SD]. Multinomial logistic regression analysis was used to examine whether fingertip AGEs predict persistent psychotic symptoms in adolescents. The significance level (α) was set to 0.05 for two-tailed tests. All statistical analyses were performed using the Statistical Package for the Social Sciences version 24.0 (IBM Corp., New York, USA).

### Reporting summary

Further information on research design is available in the Nature Research Reporting Summary linked to this article.

## Supplementary information

Supplementary Information

Reporting Summary

## Data Availability

The data that provide the findings of this study are available from the corresponding author upon reasonable request.
