# Supplementary material for: Fingertip advanced glycation end products and psychotic symptoms among adolescents
Source: NPJ Schizophr. 2021 Aug 12;7:37. doi: 10.1038/s41537-021-00167-y (PMC8361014; doi:10.1038/s41537-021-00167-y)
Supplement: Supplementary file 1 — Supplementary Information [file 41537_2021_167_MOESM1_ESM.pdf]

# Supplementary Information

**The section of materials**

Supplementary Method

Supplementary Result

Table: Supplementary Table 1, Supplementary Table 2

Figure: Supplementary Figure 1

Supplementary References

## Supplementary Method

**Validation of association between fingertip AGEs and adult psychosis**

Fifty two schizophrenia patients (9 were hospitalized) were recruited at Tokyo Metropolitan Matsuzawa Hospital, Takatsuki Hospital, and Takatsuki Clinic from September 2016 to August 2019. All patients were diagnosed according to the Diagnostic and Statistical Manual for Mental Disorders-IV criteria for schizophrenia or schizoaffective disorders by at least two experienced psychiatrists. A total of 58 age and sex matched controls were also recruited in this study. All participants with physical comorbidities, including diabetes mellitus and renal dysfunction were excluded since previous studies have indicated that these diseases affect SAF intensity[1,2]. Patients with comorbid psychiatric disorders, including alcohol dependence, illegal drug abuse, mental retardation, and neurological diseases were also excluded. This study was approved by the ethical committee of each institution, and all participants provided written informed consent.

## Supplementary Result

Participant characteristics are summarized in Supplementary Table 1. There were no significant differences in age (mean [SD], 47.3 [14.4] vs. 46.0 [7.2] years; P = .55) and sex (38% vs. 21% were male, P = .06) between schizophrenia patients and controls, while we found a significant difference in the body mass index (BMI) (mean [SD], 24.7 [4.6] vs. 22.6 [3.2] %; P = .01) between schizophrenia patients and controls. As shown in Supplementary Figure 1, the fingertip AGE levels in schizophrenia patients was significantly higher compared with that in controls (mean [SD], 0.56 [0.11] vs. 0.44 [0.05] a.u.; P<.001). A logistic regression model adjusted for age, sex, and BMI was applied to investigate the association between fingertip AGEs and schizophrenia. The analysis revealed that the OR in schizophrenia was 6.40 (95% CI: 3.02-13.55, P<.001) for a 1 SD increase in fingertip AGE level. The significance and magnitude of effect size remained after adjusting for age, sex, and BMI (OR, 5.75; 95% CI, 2.65–12.45; P<.001, Supplementary Table 2).

**Supplementary Table 1. Characteristics of adult participants in case control study**

| | **Schizophrenia (*N* = 52)** | **Control (*N* = 58)** | ***P*-value** |
|---|---|---|---|
| Fingertip AGEs (a.u., mean [SD]) | 0.56 [0.11] | 0.44 [0.05] | <.001 |
| Age (years, mean [SD]) | 47.3 [14.4] | 46.0 [7.2] | .55 |
| Sex (male/female, N) | 20 / 32 | 12 / 46 | .06 |
| BMI (kg/m$^2$, mean [SD])[1] | 24.7 [4.6] | 22.6 [3.2] | .01 |

Abbreviations: AGEs, advanced glycation end products; a.u., arbitrary unit; BMI, body mass index; CP, chlorpromazine

[1]Lack of data for 6 participants in the control group.

**Supplementary Table 2. Association between fingertip AGEs and clinical diagnosis of schizophrenia**

| | Unadjusted model | | | Adjusted model[1] | | |
|---|---|---|---|---|---|---|
| | **OR** | **95% CI** | ***P*-value** | **OR** | **95% CI** | ***P*-value** |
| Fingertip AGEs (z-scores) | 6.40 | (3.02–13.55) | <.001 | 5.75 | (2.65–12.45) | <.001 |
| Age (years) | | | | 1.01 | (0.97–1.06) | 0.516 |
| Sex | | | | 1.32 | (0.44–3.91) | 0.620 |
| BMI (kg/m$^2$) | | | | 1.14 | (0.99–1.31) | 0.068 |

Abbreviations: AGEs, advanced glycation end products; BMI, body mass index; OR, odds ratio

[1] Adjusted for age, sex, and BMI. Six cases were eliminated due to missing data on BMI.

**Supplementary Figure 1. Fingertip advanced glycation end product (AGE) levels in patients with schizophrenia and controls.**

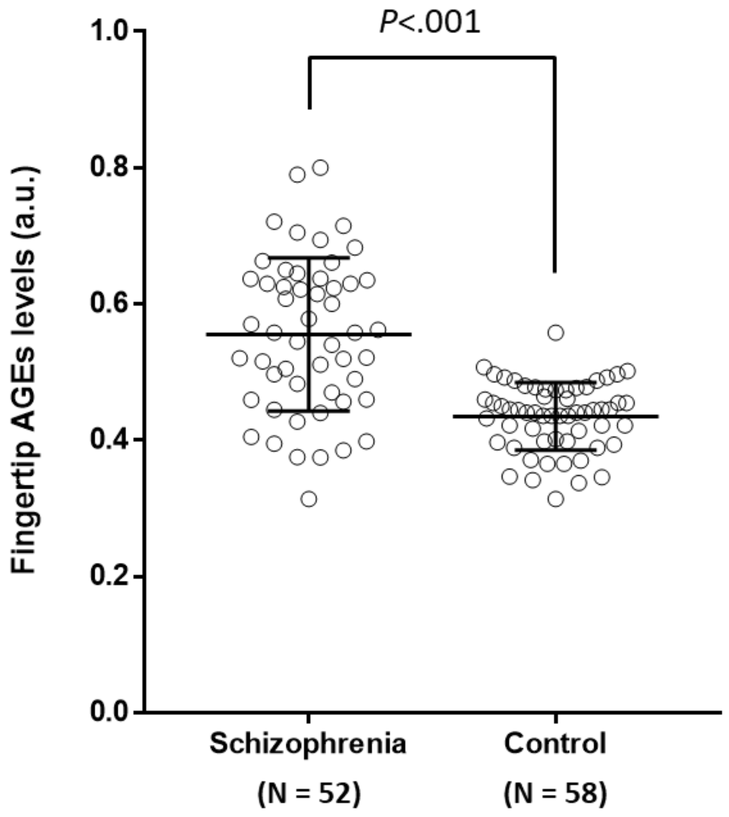


**Supplementary Figure 1.** Dot blots represent fingertip AGEs levels. Lines and error bars indicate mean and standard deviation (SD), respectively.

## Supplementary References

(1) Meerwaldt R, Graaff R, Oomen PHN, et al. Simple non-invasive assessment of advanced glycation endproduct accumulation. *Diabetologia*. 2004;47(7):1324-1330. doi: 10.1007/s00125-004-1451-2

(2) Yamanaka M, Matsumura T, Ohno R, et al. Non-invasive measurement of skin autofluorescence to evaluate diabetic complications. *J Clin Biochem Nutr*. 2016;58(2):135-140. doi: 10.3164/jcbn.15-132
