# Supplementary material for: Fingertip advanced glycation end products and psychotic symptoms among adolescents
Source: NPJ Schizophr. 2021 Aug 12;7:37. doi: 10.1038/s41537-021-00167-y (PMC8361014; doi:10.1038/s41537-021-00167-y)
Supplement: Supplementary file 2 — Reporting Summary [file 41537_2021_167_MOESM2_ESM.pdf]

nature portfolio

Corresponding author(s): DBPR, NPJSCHZ-00625

Last updated by author(s): Jun 23, 2021

Reporting Summary

Nature Portfolio wishes to improve the reproducibility of the work that we publish. This form provides structure for consistency and transparency in reporting. For further information on Nature Portfolio policies, see our Editorial Policies and the Editorial Policy Checklist.

Statistics

For all statistical analyses, confirm that the following items are present in the figure legend, table legend, main text, or Methods section.

n/a	Confirmed	
☐	☒	The exact sample size (n) for each experimental group/condition, given as a discrete number and unit of measurement
☐	☒	A statement on whether measurements were taken from distinct samples or whether the same sample was measured repeatedly
☐	☒	The statistical test(s) used AND whether they are one- or two-sided<br>*Only common tests should be described solely by name; describe more complex techniques in the Methods section.*
☐	☒	A description of all covariates tested
☐	☒	A description of any assumptions or corrections, such as tests of normality and adjustment for multiple comparisons
☐	☒	A full description of the statistical parameters including central tendency (e.g. means) or other basic estimates (e.g. regression coefficient) AND variation (e.g. standard deviation) or associated estimates of uncertainty (e.g. confidence intervals)
☐	☒	For null hypothesis testing, the test statistic (e.g. F, t, r) with confidence intervals, effect sizes, degrees of freedom and P value noted<br>*Give P values as exact values whenever suitable.*
☒	☐	For Bayesian analysis, information on the choice of priors and Markov chain Monte Carlo settings
☒	☐	For hierarchical and complex designs, identification of the appropriate level for tests and full reporting of outcomes
☐	☒	Estimates of effect sizes (e.g. Cohen's d, Pearson's r), indicating how they were calculated

Our web collection on statistics for biologists contains articles on many of the points above.

Software and code

Policy information about availability of computer code

Data collection	Not applicable for this study.
Data analysis	Not applicable for this study.

For manuscripts utilizing custom algorithms or software that are central to the research but not yet described in published literature, software must be made available to editors and reviewers. We strongly encourage code deposition in a community repository (e.g. GitHub). See the Nature Portfolio guidelines for submitting code & software for further information.

Data

Policy information about availability of data

All manuscripts must include a data availability statement. This statement should provide the following information, where applicable:

- Accession codes, unique identifiers, or web links for publicly available datasets
- A description of any restrictions on data availability
- For clinical datasets or third party data, please ensure that the statement adheres to our policy

The data that provide the findings of this study are available from the corresponding author upon reasonable request.

Field-specific reporting

Please select the one below that is the best fit for your research. If you are not sure, read the appropriate sections before making your selection.

☒ Life sciences ☐ Behavioural & social sciences ☐ Ecological, evolutionary & environmental sciences

For a reference copy of the document with all sections, see nature.com/documents/nr-reporting-summary-flat.pdf

Life sciences study design

All studies must disclose on these points even when the disclosure is negative.

Sample size	Of the 345 participants, 282 participants completed the 12 months follow up in this study.
Data exclusions	5 subjects with anti-psychotic medication were excluded from main analysis.
Replication	Not applicable for this study.
Randomization	Not applicable for the present research.
Blinding	Not applicable.

Reporting for specific materials, systems and methods

We require information from authors about some types of materials, experimental systems and methods used in many studies. Here, indicate whether each material, system or method listed is relevant to your study. If you are not sure if a list item applies to your research, read the appropriate section before selecting a response.

Materials & experimental systems

n/a	Involved in the study	
☒	☐	Antibodies
☒	☐	Eukaryotic cell lines
☒	☐	Palaeontology and archaeology
☒	☐	Animals and other organisms
☐	☒	Human research participants
☒	☐	Clinical data
☒	☐	Dual use research of concern

Methods

n/a	Involved in the study	
☒	☐	ChIP-seq
☒	☐	Flow cytometry
☒	☐	MRI-based neuroimaging

Human research participants

Policy information about studies involving human research participants

Population characteristics	The current study was conducted as part of the population-based biomarker subsample study of the Tokyo Teen Cohort Study (TTC, http://ttcp.umin.jp/) (pb-TTC), in which 345 adolescents (mean [standard deviation, SD], 13.5 [0.6] years) and their caregivers (mainly mothers) were included. The study examined biological markers, including AGEs and psychiatric symptoms as assessed with semi structured interviews by expert psychiatrists.
Recruitment	The participants in the pb-TTC were recruited from a larger sample of the TTC project, a large-scale population-based birth cohort study conducted in the Tokyo metropolitan area with > 3,000 adolescent-caregiver dyads.
Ethics oversight	The study was approved by the research ethics committees of the Tokyo Metropolitan Institute of Medical Science.

Note that full information on the approval of the study protocol must also be provided in the manuscript.
